# Ectopic HLA-II Expression in ESCC: Exploration of Its Relationship with Neoantigen Burden and Patient Survival

**DOI:** 10.3390/cells14171403

**Published:** 2025-09-08

**Authors:** Yupei Ji, Zhizhong Wang, Zhenguo Cheng, Shuangshuang Lu, Nick R. Lemoine, Renato Baleeiro, Louisa S. Chard Dunmall, Yaohe Wang

**Affiliations:** 1 Sino-British Research Centre for Molecular Oncology, National Centre for International Research in Cell and Gene Therapy, State Key Laboratory of Esophageal Cancer Prevention & Treatment, School of Basic Medical Sciences, Academy of Medical Sciences, Zhengzhou University, Zhengzhou 450001, Chinachengzhenguo@zzu.edu.cn (Z.C.); lushuangshuang@zzu.edu.cn (S.L.);; 2 Department of Molecular Pathology, Henan Provincial Tumor Hospital, Zhengzhou University, Zhengzhou 450008, China; 3 Centre for Cancer Biomarkers & Biotherapeutics, Barts Cancer Institute, Queen Mary University of London, London EC1M 6BQ, UKl.chard@qmul.ac.uk (L.S.C.D.)

**Keywords:** HLA-II, esophageal squamous cell carcinoma, neoantigen, immunohistochemistry, transcriptomics, immune microenvironment, prognosis

## Abstract

Ectopic expression of human leukocyte antigen class II (HLA-II) on tumor cells correlates with anti-tumor immunity and prognosis in various cancers, but its role in esophageal squamous cell carcinoma (ESCC) remains unclear. Methods: HLA-II expression was evaluated in 34 ESCC tissue sections and a 102-sample tissue microarray (TMA) using immunohistochemistry (IHC) and in 10 ESCC cell lines via flow cytometry. Transcriptome sequencing of KYSE270, KYSE180, KYSE450, and KYSE510 was performed to investigate HLA-II regulatory mechanisms, while tumor samples from 104 ESCC patients were analyzed for neoantigen load. The prognostic significance of neoantigen burden was assessed using Cox regression. Results: HLA-II was ectopically expressed in ESCC, with positivity rates of 20.59% (34 tissues) and 25.49% (TMA). Among 10 ESCC cell lines, only KYSE270 exhibited spontaneous HLA-II expression. Transcriptome analysis revealed 1278 KYSE270-specific genes enriched in immune-related pathways (e.g., “Cytokine–cytokine receptor interaction”), suggesting immune-mediated HLA-II regulation. IFN-γ stimulation induced HLA-II expression in KYSE180, KYSE450, and KYSE510, indicating broader inducible HLA-II potential. In 104 patients, MHC-II-restricted neoantigen burden varied widely (0–75) and lacked direct correlation with HLA-II expression. Additionally, MHC-II-restricted neoantigen load was not significantly associated with overall survival (*p* > 0.05). Conclusion: Ectopic HLA-II expression in ESCC may influence the tumor immune microenvironment, while the prognostic value of MHC-II-restricted neoantigen burden in ESCC remains unclear, providing potential implications for immunotherapy strategies.

## 1. Introduction

Esophageal squamous cell carcinoma (ESCC) is a major subtype of esophageal cancer, with high mortality and significant geographical variation in prevalence, particularly in East Asia [1,2]. Despite advancements in treatment modalities such as surgery, chemotherapy, and radiotherapy, the 5-year survival rate for ESCC remains dismally low due to delayed diagnosis and the tumor’s ability to evade immune surveillance [3,4]. Addressing the immune evasion mechanisms is critical for improving outcomes, and immunotherapy has emerged as a promising approach in cancer treatment [5]. However, in ESCC, the mechanisms by which tumors escape immune detection remain incompletely understood [6].

Human leukocyte antigen class II (HLA-II) molecules, typically expressed on professional antigen-presenting cells, such as dendritic cells, macrophages, and B lymphocytes, play a crucial role in presenting exogenous antigens to CD4+ T cells and initiating an adaptive immune response [7]. Ectopic expression of HLA-II has been observed in various tumor cells, including breast cancer [8,9], laryngeal carcinoma [10], colorectal cancer [11], melanoma [12], and pancreatic ductal adenocarcinoma (PDAC) [13]. Its expression may be induced by inflammatory cytokines such as IFN-γ and TNF-α secreted by tumor-infiltrating lymphocytes and is associated with increased CD4+ T-cell infiltration. The function of HLA-II varies across different cancers: in breast, colorectal, and laryngeal cancers, its expression correlates with favorable prognosis; whereas in melanoma, it is linked to poorer prognosis but enhances responsiveness to immune checkpoint inhibitors [14]. In PDAC, HLA-II promotes T-cell-mediated tumor cell lysis and may enhance immune recognition by presenting tumor-specific neoantigens. The role of HLA-II in tumor immunity is context-dependent, influencing the immune microenvironment and contributing to immune escape in certain malignancies [15]. However, the role of HLA-II in ESCC and its potential impact on neoantigen presentation remain largely unexplored.

This study aimed to investigate the ectopic expression of HLA-II molecules in ESCC [16] through comprehensive immunohistochemistry (IHC) [17,18], flow cytometry, and transcriptome analysis in both ESCC tissues and cell lines. In addition, we explored the relationship between HLA-II expression, neoantigen burden, and overall survival in a cohort of 104 ESCC patients, using transcriptome and exome sequencing data. Our research provides novel insights into the immunological landscape of ESCC, with potential implications for the development of targeted immunotherapies.

## 2. Materials and Methods

### 2.1. Immunohistochemistry

Paraffin-embedded tissue sections (4 μm) were prepared by the Pathology Department of the First Affiliated Hospital of Zhengzhou University. Tissue microarrays (TMAs, catalog no. HEsoS180Su08, batch no. XT18-031) were purchased from Shanghai Outdo Biotech Co., Ltd. (Shanghai, China). Sections were deparaffinized, rehydrated, and subjected to antigen retrieval in 10 mmol/L citrate buffer (pH 6.0) using a pressure cooker. Endogenous peroxidase activity was blocked with 0.45% H_2_O_2_, followed by serum blocking. MHC-II expression was detected using an anti-HLA DR + DP + DQ antibody (CR3/43) (ab7856, 1:500; Abcam, Cambridge, UK) incubated at room temperature for 1 h. A biotinylated secondary antibody was applied for 30 min, followed by detection with peroxidase-labeled streptavidin (Dako, #P0397, Agilent Technologies, Santa Clara, CA, USA) and 3,3-diaminobenzidine (Sigma-Aldrich, St. Louis, MO, USA, #D5637). Sections were counterstained with hematoxylin and mounted. Tumor cell expression was assessed by examining at least three fields at 100× magnification under a light microscope. Staining was scored based on two parameters: the percentage of positive cells and staining intensity. The percentage score was assigned as follows: 0 (0–5% positive cells), 1 (5–20%), 2 (20–40%), 3 (>40%). Staining intensity was classified as 0 (negative), 1 (background staining), 2 (weak), 3 (moderate), and 4 (strong). The final immunoreactivity score was obtained by summing the percentage and intensity scores, yielding a total score ranging from 0 to 4. We used the anti-HLA-DR + DP + DQ antibody (CR3/43, Abcam, Cambridge, UK) to simultaneously detect HLA-II subtypes (HLA-DP, HLA-DQ, HLA-DR), as this study aims to comprehensively evaluate the antigen-presenting function of HLA-II molecules in ESCC. The broad-spectrum antibody ensures capturing the expression of all HLA-II subtypes, avoiding limitations of single-subtype (e.g., HLA-DR\HLA-DP\HLA-DQ) analysis. Future studies will employ specific antibodies (e.g., anti-HLA-DR) to dissect the distinct roles of each subtype.

### 2.2. Patient Cohort

Between 2013 and 2016, a total of 120 patients diagnosed with esophageal squamous cell carcinoma (ESCC) were recruited at Anyang Cancer Hospital, Anyang, China [16]. The study was approved by the Ethics Committees of Anyang Cancer Hospital and the First Affiliated Hospital of Zhengzhou University, with written informed consent obtained from all participants. None of the patients had received radiotherapy or chemotherapy prior to surgery, and ESCC diagnosis was confirmed by three independent pathologists. Tumor tissues and adjacent normal tissues, located at least 5 cm from the tumor margin, were collected during surgical resection and stored in liquid nitrogen within 30 min post-operation. Following whole-exome sequencing, 104 patient samples with qualified sequencing data were selected for HLA-II neoantigen analysis. Demographic data, including sex and age at diagnosis, were recorded in Supplementary S1, but no sex- or age-specific analyses were performed, as these factors were not associated with the molecular profiles investigated.

### 2.3. Cell Lines

Human esophageal squamous cell carcinoma (ESCC) cell lines, including KYSE30, KYSE70, KYSE140, KYSE150, KYSE180, KYSE270, KYSE410, KYSE450, KYSE510, and KYSE520, were obtained from the Chinese Academy of Sciences Cell Bank (Shanghai, China) and maintained in our laboratory’s cell bank. Cells were cultured in RPMI 1640 medium (GIBCO, Thermo Fisher Scientific, Waltham, MA, USA) supplemented with 10% fetal bovine serum (FBS) [19,20] and these cell lines were authenticated through short tandem repeat (STR) profiling. To prevent phenotypic drift, each cell line was thawed from a new frozen vial after 50 passages. All cell lines were routinely tested for mycoplasma contamination and were only used when confirmed to be mycoplasma-free.

### 2.4. IFN-γ Stimulation

All ESCC cell lines were stimulated with 50 ng/mL human recombinant IFN-γ (#570206; BioLegend, San Diego, CA, USA) in T75 culture flasks for 48 h [21]. After incubation, the cells were harvested by trypsinization, resuspended into single-cell suspensions, and stained with purified mouse IgG antibodies targeting HLA-DR, DP, and DQ (clone Tü39; #555556; BD, Herlev, Denmark). Secondary antibody incubation was performed with Alexa Fluor 488-conjugated anti-mouse IgG. The cells were then analyzed by flow cytometry.

### 2.5. Prediction of Potential Neoepitopes from ESCC Patients

Potential neoepitopes were identified from the whole-exome sequencing (WES) data of 104 confirmed ESCC patients [16] using pVACtools 4.2 [22]. Germline mutations were detected from matched normal tissue sequencing data using the GATK Haplotype Caller (4.0.6.0) and CNN Score Variants tools (GATK 4). Somatic variants detected by Varscan2 were downloaded, annotated with the Ensembl Variant Effect Predictor (110) [23], and integrated with gene expression data. A phased VCF file was generated by combining germline and somatic variant calls using GATK Combine Variants and Read Backed Phasing tools (GATK 4). Patient-specific HLA haplotypes were determined using Seq2HLA (2.2) [24] and used as input for the pVACtools 4.2 pvacseq [25] module. Candidate neoepitopes were filtered based on median binding affinity and DNA variant allele frequency (minimum 0.25).

### 2.6. Flow Cytometry

Cell surface labeling was performed by incubating cell suspensions with specific antibodies for 30 min at room temperature. For intracellular staining, cells were first fixed and permeabilized using the Leucoperm kit (Bio-Rad, Hercules, CA, USA; #BUF09C), in accordance with the manufacturer’s instructions. To assess IFN-γ production, cells were incubated overnight in the presence of 5 μg/mL brefeldin A (Sigma-Aldrich; #B6542-5 MG) before staining. Cell viability was determined by dual staining with Ethidium homodimer 1 and Calcein-AM, using the LIVE/DEAD^®^ Viability/Cytotoxicity Kit (Invitrogen, Carlsbad, CA, USA; #L3224), according to the manufacturer’s protocol.

Flow cytometric analysis was carried out using either a FACSCalibur (Becton Dickinson, Heidelberg, Germany) or BD LSRII (Becton Dickinson) flow cytometer. Data were processed using FlowJo software (version 10.8.1), and further statistical analysis and visualization were conducted using Microsoft Excel and Python.

### 2.7. Whole-Exome Sequencing (WES) Experiment

Genomic DNA was extracted from tumors and matched normal samples or peripheral blood using the QIAamp DNA Mini Kit (Qiagen, Venlo, The Netherlands) following the manufacturer’s protocol. For whole-exome capture library construction, 1 μg of genomic DNA from each fresh-frozen tumor and matched normal sample was fragmented into 250–300 bp using Covaris. Fragments were purified with the AxyPrep Mag PCR Clean-up Kit (Corning, New York, NY, USA) and captured using the Agilent SureSelect Human Exomes V6 kit (~35.7 Mb, Cat No.: 51908881). Libraries were sequenced on the Illumina X Ten platform (Illumina, San Diego, CA, USA), generating 150 bp paired-end reads [16].

### 2.8. Whole-Exome Sequencing (WES) Data Analysis

After quality control with FastqQC (0.11.7), reads were aligned to the hg19 genome using BWA (0.7.17) mem, with alignments refined by GATK4 (4.0.6.0) per best practice guidelines. Somatic variants were identified using Mutect2 and Strelka2 (2.8.4), retaining only variants detected by both. Variants were annotated with Ensembl Variant Effect Predictor [16].

### 2.9. RNA-Seq Experiment

Total RNA was isolated from tumors and matched normal samples using Invitrogen’s TRIzol reagent following the manufacturer’s protocol. RNA was quantified with the Agilent 2100 Bioanalyzer (Agilent RNA 6000 Nano Kit), and 1 μg of RNA was used to prepare sequencing libraries with the VAHTS^®^ Total RNA-Seq (H/M/R) Library Prep Kit for Illumina^®^ (San Diego, CA, USA). Quantified libraries were sequenced on the Illumina X Ten platform (Illumina, San Diego, CA, USA) with 150 bp paired-end reads, yielding an average of 120 million reads per sample [16].

### 2.10. RNA-Seq Data Analysis

Raw reads were quality-checked using FastQC (0.11.7). Clean reads, filtered by SOAPnuke 1.5.6 (-l 10 -q 0.5 -n 0.05 -Q 2 -G), were aligned and quantified against the GRCh37 genome using Salmon (0.9.0). Transcriptome abundances were imported into R (3.5.1) via the “tximport” package. Transcripts with TPM > 1 in over half of the samples were retained and normalized using cqn (R3.5.1). Principal component analysis (PCA) was performed on the transcriptome to assess data quality and potential biases. Differential expression analysis for tumor versus normal pairs was conducted using Limma (3.38.3) [16].

### 2.11. HLA Typing

Seq2HLA software(2.2) was used for HLA typing and expression analysis based on RNA-Seq data, following the workflow below. Data input: Raw RNA-Seq data in fastq format were used as input, containing transcriptome sequencing reads of samples. Sequence alignment: Sequencing reads were aligned to the HLA reference database using a bowtie index containing all known HLA alleles to locate specifically matched reads. Preliminary allele screening: For each locus of HLA class I (A, B, C) and class II (DRB1, DQA1, DQB1, etc.), the allele with the highest coverage was selected as a candidate. Iterative validation: After removing reads matched to the preliminarily screened alleles, re-alignment was performed to identify the second allele at the same locus (for heterozygous cases). Genotype determination: The homozygous/heterozygous status of each locus was determined by combining results from two iterations, and finally HLA genotypes with 4-digit resolution, confidence *p*-values, and expression levels of each allele were output. To ensure analytical reliability, FastQC was used for quality control of raw RNA-Seq data prior to seq2HLA(2.2) analysis, evaluating metrics such as sequence quality distribution, base composition, adapter contamination, and proportion of duplicate sequences [24].

### 2.12. Neoantigen Prediction

Filtered somatic variants (variant allele frequency ≥ 0.25) and patient HLA genotypes were input into the pvacseq module of the pVACtools to predict MHC-II-restricted neoantigens. Screening criteria include: median binding affinity (IC50 < 500 nM) and peptide length matching HLA-II binding characteristics (typically 15–20 amino acids) [22] (https://pvactools.readthedocs.io/en/latest/pvacseq/getting_started.html 15 March 2025).

### 2.13. Statistical Analysis

All statistical analyses were performed using R (4.0.2) and Python (3.8). Prior to data analysis, all datasets (including clinical data from 104 ESCC patients such as survival time, survival status, age, sex, and pTNM stage, as well as molecular data such as neoantigen count and HLA-II expression levels) were standardized to ensure consistency in the analysis. Standardization was achieved by converting continuous variables into z-scores (mean = 0, variance = 1), with missing values checked and excluded (e.g., one patient with missing survival time resulted in a final sample of 103).

To compare expression differences between different samples, independent *t*-tests were used, with normality and homogeneity of variances tested before applying parametric tests. To investigate the relationship between HLA-II expression and patient survival, the Cox proportional hazards model was employed, with the number of neoantigens treated as a covariate to assess its potential impact on survival time. The proportional hazards assumption was validated for all models. Survival curves were generated using the Kaplan–Meier method, and differences between groups were compared using the log-rank test to evaluate survival time differences across different neoantigen burden groups.

We used the Kaplan–Meier method to generate survival curves and compared the survival time differences between groups with different neoantigen burdens using the log-rank test. To clarify the grouping criteria, we based our classification on the neoantigen counts of 103 ESCC patients (ranging from 0 to 75 neoantigens per sample) and used the median value (9 neoantigens per sample) as the threshold to divide the samples into two groups: the high neoantigen burden group (≥9 neoantigens, *n* = 53) and the low neoantigen burden group (<9 neoantigens, *n* = 50). We have supplemented the Kaplan–Meier curves (Supplementary S2), which show no significant survival differences between the two groups (log-rank *p* = 0.80). Initially, 104 patients were included, but 1 patient was excluded due to missing survival time data caused by technical issues, resulting in the final analysis being based on 103 patients. Additionally, the scatter plot analyzed the relationship between neoantigen count and survival time using linear regression and Pearson correlation to assess linear association.

To assess the correlation between neoantigen count and HLA-II expression levels, Spearman’s rank correlation coefficient was used. All statistical tests were conducted with a significance threshold of *p* < 0.05. For multiple comparisons, Bonferroni correction was applied to adjust *p*-values, ensuring that the overall type I error rate was controlled [26,27,28].

Additionally, principal component analysis (PCA) and clustering analysis techniques were applied to explore potential patterns and groupings within the data, helping to identify underlying differences and classifications between samples. (SEM data are presented as mean ± standard error). *p* < 0.05 was considered statistically significant (* *p* < 0.05; ** *p* < 0.01; *** *p* < 0.001; **** *p* < 0.0001). These data were generated as part of a prior study conducted by our group and have been previously published in (https://www.nature.com/articles/s41467-024-53164-x 1 March 2025) [16].

## 3. Results

### 3.1. Ectopic Expression of HLA-II Molecules in Human Esophageal Squamous Cell Carcinoma

**Figure 1:**
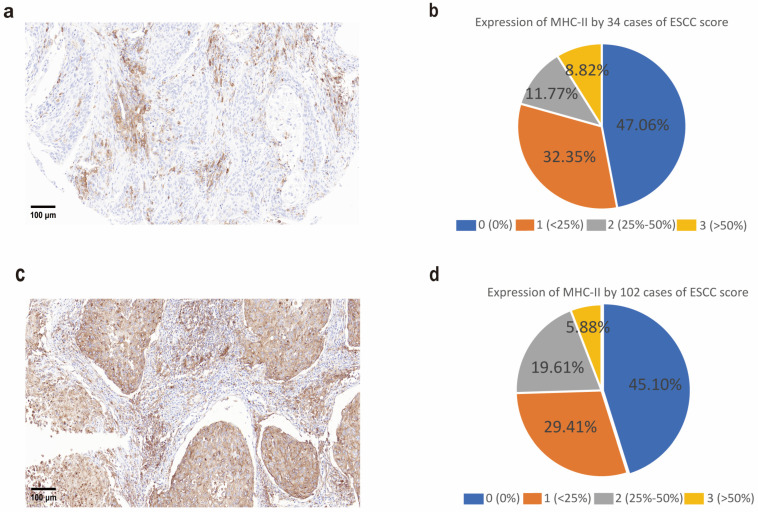
HLA-II is ectopically expressed in ESCC. (**a**) Representative images of HLA-II immunohistochemical (IHC) staining in ESCC tissues. HLA-II positive staining is shown in brown. (**b**) Quantification of HLA-II IHC staining in 34 ESCC tissues. The pie chart shows the percentage of samples with different HLA-II staining intensity scores (0–3). (**c**) Representative images of HLA-II IHC staining in a tissue microarray (TMA) containing 180 samples, including 102 ESCC tumor tissues and 66 normal esophageal tissues. HLA-II positive staining is shown in brown. (**d**) Quantification of HLA-II IHC staining in 102 ESCC tumor tissues from the TMA. The pie chart shows the percentage of samples with different HLA-II staining intensity scores (0–3).

To investigate the expression of HLA-II molecules in esophageal squamous cell carcinoma (ESCC), we first performed immunohistochemical (IHC) staining on tumor tissue sections from 34 ESCC patients. The results revealed HLA-II expression in 7 out of 34 samples (20.59%), with scores of 2 and 3 defined as positive and a score of 1 defined as negative (Figure 1a,b). To further validate HLA-II expression, we conducted IHC staining on a tissue microarray containing 180 samples, including 102 ESCC tissues (114 ESCC in total, 12 samples were excluded due to tissue detachment) and 66 non-tumor esophageal tissues. The results showed that HLA-II was expressed in 26 (25.49%) of the 102 ESCC tissue samples (Figure 1c,d). Through these two independent validations using small-scale samples and a tissue microarray, we confirmed the ectopic expression of HLA-II in ESCC, laying the foundation for subsequent studies.

### 3.2. Ectopic Expression of HLA-II Molecules in ESCC Cell Lines

**Figure 2:**
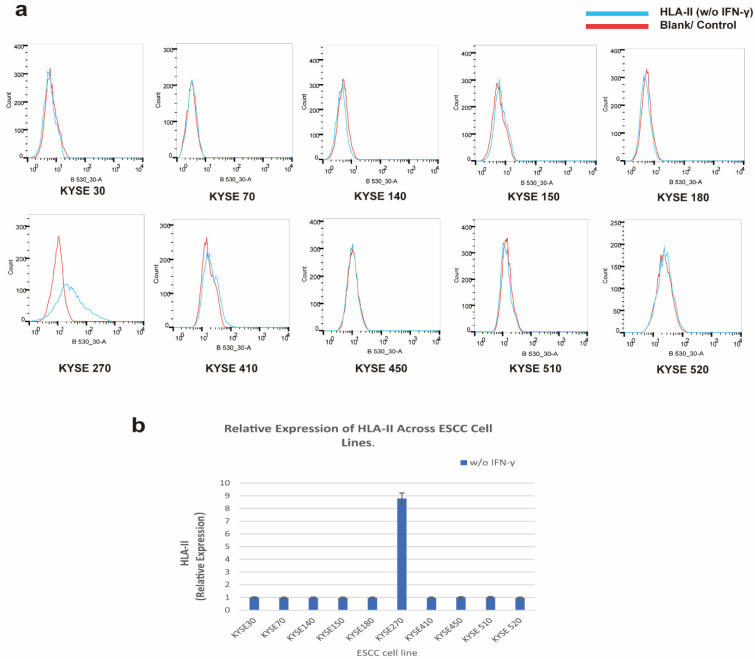
Ectopic Expression of HLA-II in ESCC Cell Lines. (**a**) Flow cytometry analysis of HLA-II expression in 10 ESCC cell lines. The blue peak represents HLA-II expression without IFN-γ stimulation, i.e., spontaneous expression (HLA-II (*w*/*o* IFN-γ)), and the red peak represents the blank control (Blank/Control). (**b**) Quantification of HLA-II expression in ESCC cell lines. The relative expression level was normalized to the blank control group of the KYSE 30 cell line (i.e., the HLA-II expression of the Blank group of KYSE 30 was set as 1); the experiment was performed with 3 biological replicates, and the data are presented as mean ± standard deviation.

To explore HLA-II expression at the cellular level in ESCC, we examined 10 ESCC cell lines (KYSE30, KYSE70, KYSE140, KYSE150, KYSE180, KYSE270, KYSE410, KYSE450, KYSE510, KYSE520) for HLA-II expression. Flow cytometry analysis revealed that only KYSE270 cells spontaneously expressed HLA-II, while no HLA-II expression was detected in the other cell lines without stimulation (Figure 2a,b). This finding indicates that KYSE270 exhibits a unique characteristic of spontaneous HLA-II expression, providing an ideal cellular model for further mechanistic studies.

### 3.3. Transcriptomic Characteristics and IFN-γ Stimulation Reveal Mechanisms of HLA-II Expression in ESCC

To explore the mechanism underlying the spontaneous expression of HLA-II in KYSE270 cells, we performed transcriptome sequencing on KYSE270, KYSE180, KYSE450, and KYSE510 cells and analyzed differentially expressed genes (DEGs). For transcriptomic analysis, KYSE270 was chosen as the reference control for DEG because it is the only cell line among KYSE270, KYSE180, KYSE450, and KYSE510 that spontaneously expresses HLA-II, as confirmed by flow cytometry.

**Figure 3:**
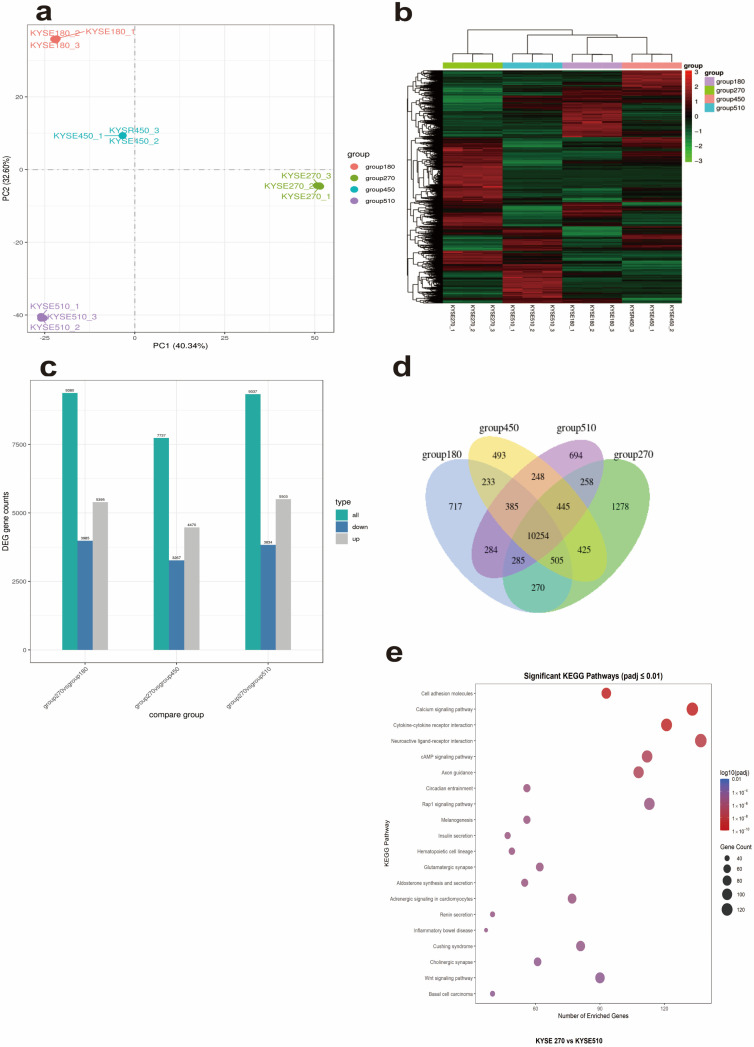
Transcriptome sequencing analysis of KYSE270 and other ESCC cell lines. (**a**) PCA Analysis of Transcriptome. Principal component analysis (PCA) plot illustrating the overall transcriptome differences among ESCC cell lines (KYSE180, KYSE270, KYSE450, KYSE510). Each dot represents a biological replicate, and separation along PC1 (40.34%) and PC2 (32.60%) reflects inter-cell-line transcriptional variation. KYSE270 samples cluster distinctly, indicating unique expression signatures. (**b**) Hierarchical Clustering of DEGs. Heatmap of differentially expressed genes (DEGs) across ESCC cell lines, generated by hierarchical clustering. Red and green represent high and low expression, respectively. KYSE270 shows clustered separation from other cell lines (KYSE180, KYSE450, KYSE510), highlighting cell-line-specific transcriptional programs. (**c**) Number of differentially expressed genes (DEGs) between KYSE270 and other ESCC cell lines (KYSE180, KYSE450, KYSE510). DEGs were identified using a |log2 fold change| ≥ 1 and adjusted *p*-value (padj) < 0.05. (**d**) Venn diagram showing the overlap of highly expressed genes (TPM > 1) between KYSE270 and other ESCC cell lines (KYSE180, KYSE450, KYSE510). (**e**) KEGG enrichment analysis of all DEGs between KYSE270 and KYSE510. The dot plot shows enriched pathways, with dot size representing gene count and color indicating adjusted *p*-value (padj).

A large number of DEGs were identified between KYSE270 and the other three cell lines, with similar numbers of upregulated and downregulated genes (Figure 3a). Venn diagram analysis revealed 1278 KYSE270-specific genes (TPM > 1), including multiple HLA-II family genes (e.g., *HLA-DRB5*, *HLA-DQA2*, *HLA-DMB*) and immunoglobulin superfamily genes (e.g., OSCAR, IGLV1-51), suggesting distinct immune activity in KYSE270 (Figure 3b). Notably, HLA-DRB5 expression in KYSE270 reached TPM = 50, compared to TPM < 1 in KYSE180, KYSE450, and KYSE510, consistent with its spontaneous HLA-II expression. KEGG enrichment analysis of all DEGs between KYSE270 and KYSE510 (including the 1278 specific genes) showed significant enrichment in immune-related pathways, such as “Cytokine–cytokine receptor interaction” and “Cell adhesion molecules” (padj < 0.01, Figure 3c), supporting KYSE270’s HLA-II-positive phenotype. Other enriched pathways, like “Basal cell carcinoma” and “Wnt signaling pathway,” may reflect additional tumor-related features of KYSE270. Similar immune-related pathways were enriched in comparison with KYSE180 and KYSE450.

### 3.4. IFN-γ Stimulation Validates the Regulatory Mechanisms of HLA-II Expression

**Figure 4:**
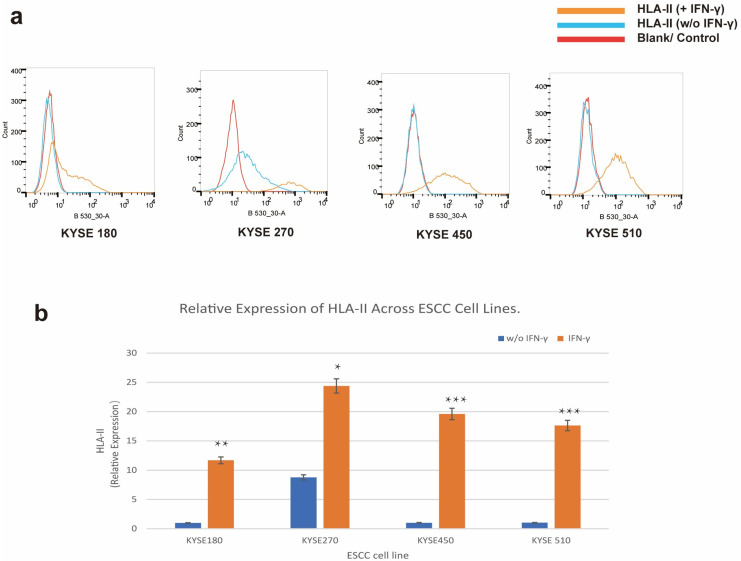
IFN-γ Stimulation Validates HLA-II Expression Regulation. (**a**) Flow cytometry analysis of HLA-II expression in KYSE180, KYSE270, KYSE450, and KYSE510 with or without IFN-γ stimulation. Histograms show fluorescence intensity (B530_30-A) for HLA-II expression. (**b**) Quantification of HLA-II expression in KYSE180, KYSE270, KYSE450, and KYSE510 with or without IFN-γ stimulation. The bar chart represents the relative expression of HLA-II, normalized to the KYSE180 *w*/*o* IFN-γ group. Error bars indicate the standard deviation (SD) from three independent experiments. Statistical significance was determined by comparing the *w*/*o* IFN-γ and + IFN-γ groups using a two-tailed *t*-test. Significance levels are indicated as follows: * *p* < 0.05, ** *p* < 0.01, *** *p* < 0.001.

To validate the functional significance of the transcriptomic findings, we performed IFN-γ stimulation experiments on KYSE180, KYSE450, and KYSE510 cells. Flow cytometry analysis showed that IFN-γ stimulation induced HLA-II expression in KYSE180, KYSE450, and KYSE510 cells, while KYSE270 maintained high HLA-II levels with or without IFN-γ stimulation (Figure 4a,b). These results suggest that the immune-related pathways identified through transcriptomic sequencing, such as “Cytokine–cytokine receptor interaction,” may regulate HLA-II expression via IFN-γ signaling, providing further evidence for the mechanisms of HLA-II ectopic expression.

### 3.5. Capability of ESCC Tumors to Generate MHC Class II Neoepitopes

**Figure 5:**
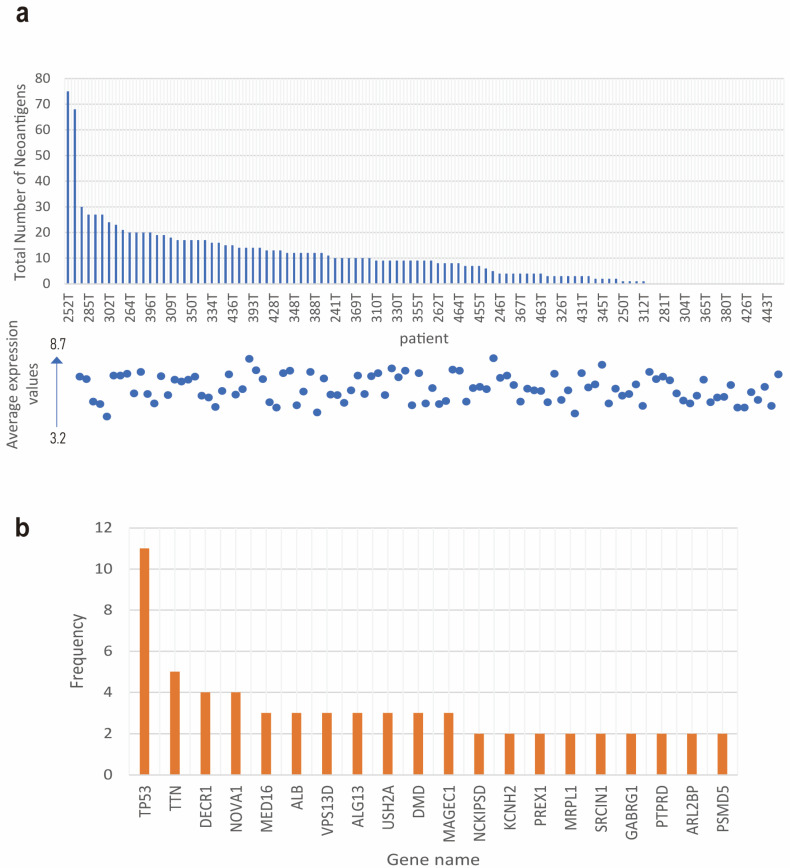
Landscape of neoantigens in ESCC patients. (**a**) Total number of neoantigens predicted for each of the 104 ESCC patients. The dot plot graph shows the average expression values of HLA-II. (**b**) Top 20 most frequently mutated genes in 104 ESCC patients.

The ectopic expression of HLA-II molecules in esophageal squamous cell carcinoma (ESCC) suggests a potential role in immune regulation through the presentation of neoantigens within the tumor microenvironment. To further investigate the functional expression of these HLA-II molecules, we conducted a neoantigen prediction analysis specifically for HLA-DPB1*05:01-restricted neoantigens [29] using transcriptome and exome data from 104 ESCC patients [16]. As shown in Figure 5a, there was significant variation in the number of neoantigens among patients, ranging from 0 to 75 with an average of 10.09 and a median of 9. Most neoantigens exhibited high binding affinity to HLA-II molecules (IC50 < 500 nM), and a negative correlation was noted between the hydrophobicity of the neoantigens and their binding affinity. Interestingly, no significant correlation was found between the predicted number of neoantigens and the expression levels of HLA-II molecules. Further analysis of the mutational genes producing these neoantigens (Figure 5b) revealed high mutation frequencies in genes such as *TP53*, *TTN*, *MUC16*, *CSMD3*, *LRP1B*, *RYR2*, *ZFHX4*, *DNAH5*, *SYNE1*, *PCLO*, *FLG*, *USH2A*, *FAT3*, and *CSMD1*. Mutations in these genes may contribute to increased neoantigen production, potentially triggering stronger anti-tumor immune responses in ESCC patients. Overall, these findings suggest that ESCC patients may generate a large number of new HLA-II epitopes, which could play a crucial role in the anti-tumor immunity in ESCC and serve as potential targets for immunotherapeutic strategies.

To analyze the correlation between the expression levels of *HLA-DP*\*DQ*\*DR* genes and the number of neoantigens, first, the neoantigen data (neoantigen_analysis_sorted_ic50_with_sequences.csv, Supplementary S4) and the HLA-DP\DQ\DR gene expression data (HLA-DP logFold.txt, Supplementary S5) were matched by sample numbers (e.g., “252T” corresponds to “ESC00000000252T”), and finally 78 common samples were obtained. The average expression values of the core genes in the HLA-DP\DQ\DR group (HLA-DPA1, HLA-DPB1, HLA-DRB1, HLA-DQA1, HLA-DQB1) in the samples were selected as the overall expression level of HLA-DP\DQ\DR. The average expression values (ranging from 3.2 to 8.7) were used as the y-axis values to replace HLA-II RPKM, and correlation coefficient was used to analyze the association between this overall expression level and the number of neoantigens. The results showed that there was no significant correlation between the two (*r* = 0.083, *p* = 0.476), supporting the conclusion that the expression levels of HLA-DP\DQ\DR genes are not related to the number of neoantigens.

### 3.6. Relationship Between Neoantigen Burden and Survival in Esophageal Cancer

**Figure 6:**
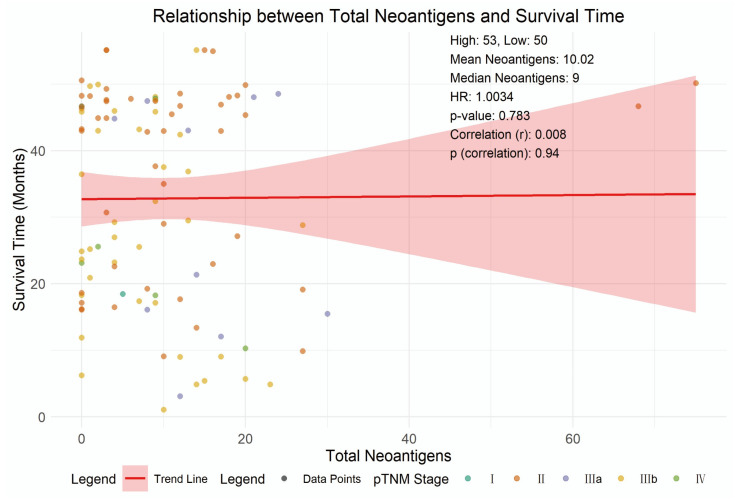
Relationship between total neoantigens and survival time. Scatter plot shows the relationship between the total number of neoantigens and survival time in 103 ESCC patients. The analysis was performed using Cox regression with only complete data records. The red line represents the trend line. The shaded area represents the 95% confidence interval.

To evaluate the impact of neoantigen burden on survival, we divided 103 ESCC patients into high (≥9 neoantigens, *n* = 53) and low (<9 neoantigens, *n* = 50) neoantigen burden groups based on the median neoantigen count. Kaplan–Meier analysis showed no significant survival difference (log-rank *p* = 0.80, Supplementary S2). A Cox proportional hazards model, adjusted for age, sex, and pTNM stage, indicated that neoantigen burden was not significantly associated with survival (HR = 1.003, 95% CI: 0.979–1.028, *p* = 0.783, Figure 6). Advanced pTNM stage II (vs. stage I) was a significant predictor (HR = 4.25, 95% CI: 1.014–17.817, *p* = 0.048), while age, sex, and other pTNM stages were not significant predictors (Supplementary S1). A scatter plot showed the relationship between neoantigen count and survival time, with a trend line and a Pearson correlation coefficient (*r* ≈ 0, *p* > 0.05) indicating a very low correlation (Figure 6). Initially, 104 patients were included, but 1 patient was excluded from the final analysis of 103 due to missing survival time data caused by a technical issue.

## 4. Discussion

Our study provides a comprehensive investigation into the ectopic expression of HLA-II in esophageal squamous cell carcinoma (ESCC) and its association with neoantigens and prognosis. We observed ectopic expression of HLA-II in ESCC tissues and cell lines [30], with KYSE270 cells uniquely showing spontaneous HLA-II expression, while KYSE180, KYSE450, and KYSE510 expressed HLA-II following IFN-γ stimulation. This suggests that HLA-II expression may be regulated by immune signals in the tumor microenvironment, and KYSE270 may possess a distinct HLA-II expression mechanism [31,32]. However, IHC analysis indicated that a significant proportion of HLA-II expression may originate from tumor-associated macrophages (TAMs) rather than tumor cells themselves, aligning with prior findings that macrophages are key antigen-presenting cells expressing MHC class II in cancers. These findings, validated by pathologists from our institution’s Pathology Department, suggest TAMs may contribute to HLA-II expression in ESCC. Further studies are needed to quantify the cellular sources of HLA-II expression.

Transcriptome sequencing revealed 1278 KYSE270-specific genes, including HLA-II family genes (e.g., HLA-DRB5), and all differentially expressed genes between KYSE270 and KYSE510 were enriched in immune-related pathways like “Cytokine–cytokine receptor interaction” (Figure 3c). These pathways may contribute to the spontaneous HLA-II expression in KYSE270 and potentially to immune escape mechanisms in ESCC. To clarify the association between KYSE270-specific genes and immune regulatory pathways, we performed protein–protein interaction (PPI) enrichment analysis on 1278 KYSE270-specific genes using Metascape. The results showed that the gene set was significantly enriched in immune-related functional modules and pathways (see Supplementary S3 Material and https://metascape.org/gp/index.html#/reportfinal/tfxxm2nnq 19 June 2025 for full analysis results). Specifically, the MCODE_4 module was enriched in the JAK-STAT signaling pathway (hsa04630, Log_10_(P) = −13.6), suggesting that highly expressed genes in KYSE270 can synergistically participate in JAK-STAT pathway regulation through the PPI network; meanwhile, the MCODE_7 module was enriched in the non-canonical NF-κB pathway (e.g., TNFR2 non-canonical NF-κB pathway, Log_10_(P) = −12.8), indicating a functional association between specific genes and the NF-κB pathway.

Given the known functions of these pathways—JAK-STAT signaling modulates anti-tumor immune responses, and NF-κB is involved in inflammation and immune regulation —we hypothesize that they may synergistically affect HLA-II expression in KYSE270 and the tumor microenvironment. Subsequent functional experiments (e.g., pathway inhibitor intervention, gene knockdown/overexpression) can verify their regulatory mechanisms on HLA-II expression, providing a basis for exploring immunotherapeutic targets in ESCC. Previous studies have shown that JAK-STAT signaling [33] modulates anti-tumor immune responses, while NF-κB is closely associated with inflammation and immune regulation [34,35]. These findings suggest that JAK-STAT and NF-κB pathways may serve as potential therapeutic targets in ESCC, such as through JAK or NF-κB inhibitors to modulate HLA-II expression and enhance anti-tumor immunity.

We predicted HLA-DPB1*05:01-restricted neoantigens in 104 ESCC patients, finding significant variation in neoantigen numbers. Notably, no direct correlation was observed between neoantigen load and HLA-II expression levels. This lack of correlation may be attributed to several factors. First, HLA-II expression in ESCC tissues may predominantly occur in TAMs rather than tumor cells, as suggested by our IHC results, meaning that HLA-II expression levels may not directly reflect the tumor cells’ capacity to present neoantigens. Second, neoantigen load is likely influenced by tumor mutation burden (TMB) and the efficiency of antigen processing and presentation machinery (e.g., TAP, tapasin, and immunoproteasome components), which are independent of HLA-II expression levels. Third, our study did not perform experimental validation (e.g., in vitro neoantigen presentation assays or T-cell activation studies) to confirm the functional presentation of predicted neoantigens by HLA-II molecules, which limits our ability to establish a direct link between HLA-II expression and neoantigen load. Future studies should incorporate functional assays to validate neoantigen presentation and explore additional factors influencing neoantigen generation in ESCC. Nevertheless, we hypothesize that the heterogeneity in HLA-II expression may indirectly influence the immune microenvironment by modulating neoantigen presentation efficiency. For instance, tumor cells with high HLA-II expression (e.g., KYSE270) may more effectively activate CD4+ T cells, enhancing immune responses against neoantigens and thereby improving the patient’s immune status.

To evaluate the impact of neoantigen burden on survival, we performed Kaplan–Meier analysis and Cox proportional hazards regression in 103 ESCC patients (1 excluded due to missing survival data). Patients were stratified into high (≥9 neoantigens, *n* = 53) and low (<9 neoantigens, *n* = 50) burden groups based on the median neoantigen count. Kaplan–Meier analysis showed no significant difference in overall survival between the two groups (log-rank *p* = 0.80, Supplementary S2). A Cox model adjusted for age, sex, and pTNM stage further confirmed that neoantigen burden was not significantly associated with survival (HR = 1.003, 95% CI: 0.979–1.028, *p* = 0.783, Figure 6). Among other variables, only advanced pTNM stage II (vs stage I) was a significant predictor of survival (HR = 4.25, 95% CI: 1.014–17.817, *p* = 0.048), while age, sex, and other stages showed no significant associations (Supplementary S1). A scatter plot with a trend line and Pearson correlation analysis (r ≈ 0, *p* > 0.05) further indicated a negligible linear relationship between neoantigen count and survival time (Figure 6). Notably, our findings differ from previous reports linking higher neoantigen burden to better prognosis in other cancers. Potential explanations for this discrepancy include the unique immunological features of ESCC (e.g., immunosuppressive tumor microenvironment limiting neoantigen recognition), the focus on HLA-II-restricted neoantigens specifically, or the moderate sample size. Further studies with larger cohorts and comprehensive assessment of neoantigen quality (e.g., binding affinity, immunogenicity) may clarify the role of neoantigen burden in ESCC survival.

This study has limitations. Our sample size was relatively small, and future studies with larger cohorts are needed to validate our findings. Moreover, we only predicted neoantigens restricted to HLA-DP, and neoantigens for other HLA alleles remain to be explored. Additionally, the lack of experimental validation for neoantigen presentation limits our conclusions regarding the functional role of HLA-II in ESCC. Furthermore, there is a discrepancy between the cell line data (KYSE cell lines) and patient data in this study. While KYSE cell lines provide a homogeneous experimental model for elucidating the molecular mechanisms of HLA-II expression, they cannot fully recapitulate the complexity of the patient tumor microenvironment, such as the absence of in vivo cellular interactions. Future studies could perform transcriptomic sequencing on patient samples to further validate the relationship between HLA-II expression mechanisms, neoantigen burden, and prognosis.

Future studies could explore the correlation between HLA-DR\DP\DQ expression and tumor immunosuppressive mechanisms, such as regulatory T cells (Tregs), myeloid-derived suppressor cells (MDSCs), and type I macrophages, to elucidate their roles in countering neoantigen load and host immunity. Our findings suggest that HLA-II expression may partially originate from tumor-associated macrophages, providing preliminary evidence for studying HLA-DR\DP\DQ’s interaction with immunosuppression. Subsequent studies will assess HLA-DR\DP\DQ co-expression with Tregs and MDSCs via single-cell RNA sequencing or immunofluorescence and evaluate combining immune checkpoint inhibitors (e.g., anti-PD-1/PD-L1) in ESCC immunotherapy.

In conclusion, our findings highlight the ectopic expression of HLA-II in ESCC and its relevance to neoantigen presentation and patient prognosis, providing a foundation for developing HLA-II-targeted immunotherapies in ESCC. The novelty of this study lies in its integrative approach, combining tissue expression, cellular mechanisms, and neoantigen analysis to systematically investigate the roles of HLA-II and neoantigens in ESCC. Future research could explore targeted therapeutic strategies, such as JAK-STAT or NF-κB pathway inhibitors, in combination with neoantigen prediction, to develop combined immunotherapeutic approaches, further optimizing treatment outcomes for ESCC patients.

## Data Availability

The sequence data used in this study were obtained from the National Genomics Data Centre of China (NGDC) at [https://bigd.big.ac.cn/bioproject/browse/PRJCA001577 (accessed on 15 May 2021)], BioProject ID PRJCA001577. The raw sequencing data of RNA-seq and whole exome sequencing are accessible under GSA accession numbers HRA000111 and HRA000112, respectively. The availability of the data has been approved by the Human Genetic Resources Registration System of the Ministry of Science and Technology of the People’s Republic of China with the registration number 2024BAT00864. HLA-II neoantigen analysis was performed on these data as part of this study.

## Supplementary Materials

The following supporting information can be downloaded at: https://www.mdpi.com/article/10.3390/cells14171403/s1.
