# Supplementary figures and images for: Ectopic HLA-II Expression in ESCC: Exploration of Its Relationship with Neoantigen Burden and Patient Survival
Source: Cells. 2025 Sep 8;14(17):1403. doi: 10.3390/cells14171403 (PMC12427808; doi:10.3390/cells14171403)

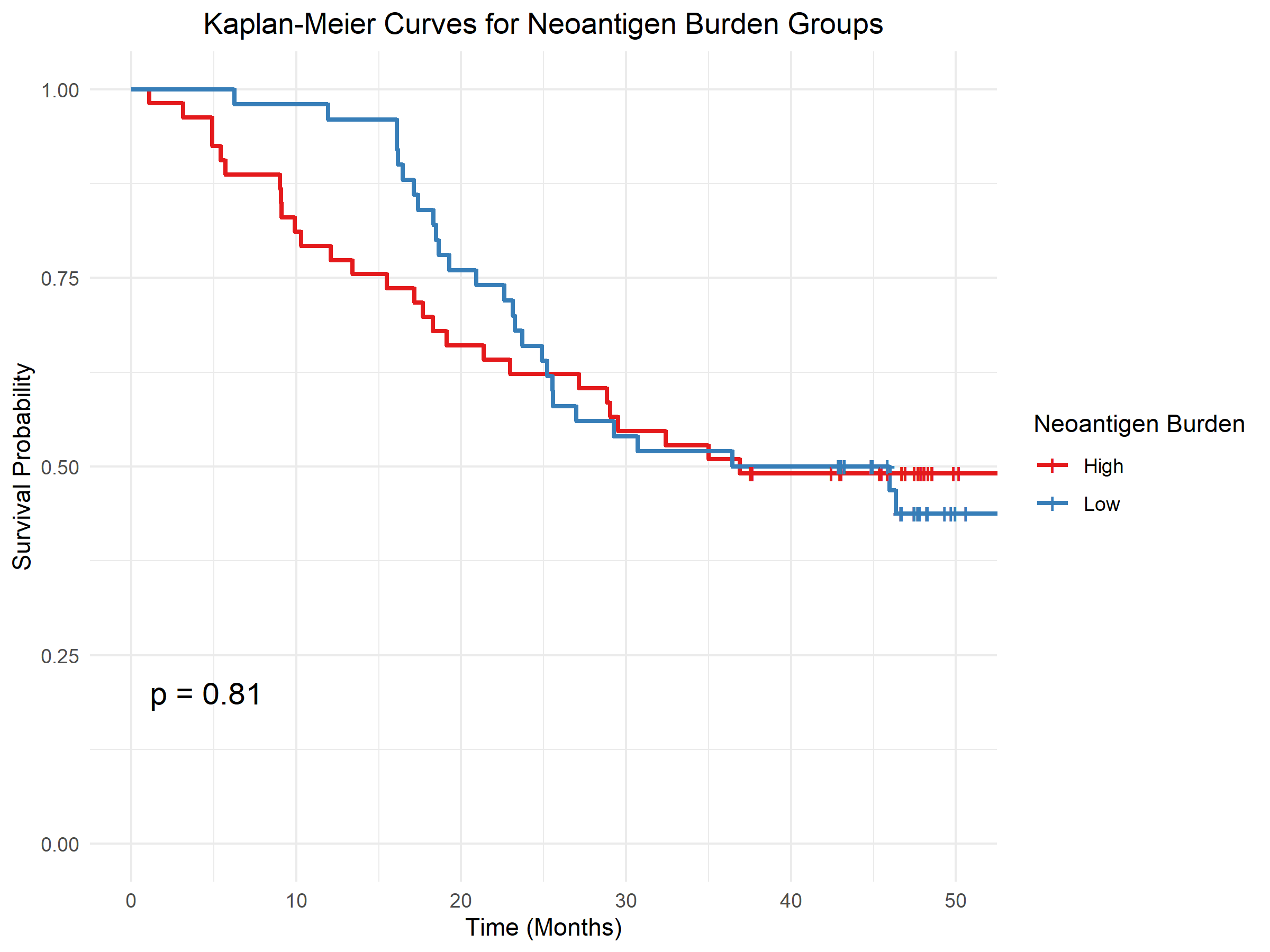

Supplement: Supplementary file 1 [file cells-14-01403-s001.zip › Supplementary S2 kaplan -Meier.png]
