# Supplementary material for: Ectopic HLA-II Expression in ESCC: Exploration of Its Relationship with Neoantigen Burden and Patient Survival
Source: Cells. 2025 Sep 8;14(17):1403. doi: 10.3390/cells14171403 (PMC12427808; doi:10.3390/cells14171403)
Supplement: Supplementary file 1 [file cells-14-01403-s001.zip › Supplementary S3_group270 list.zip!/Supplementary S3_PPI_group 270.pdf]

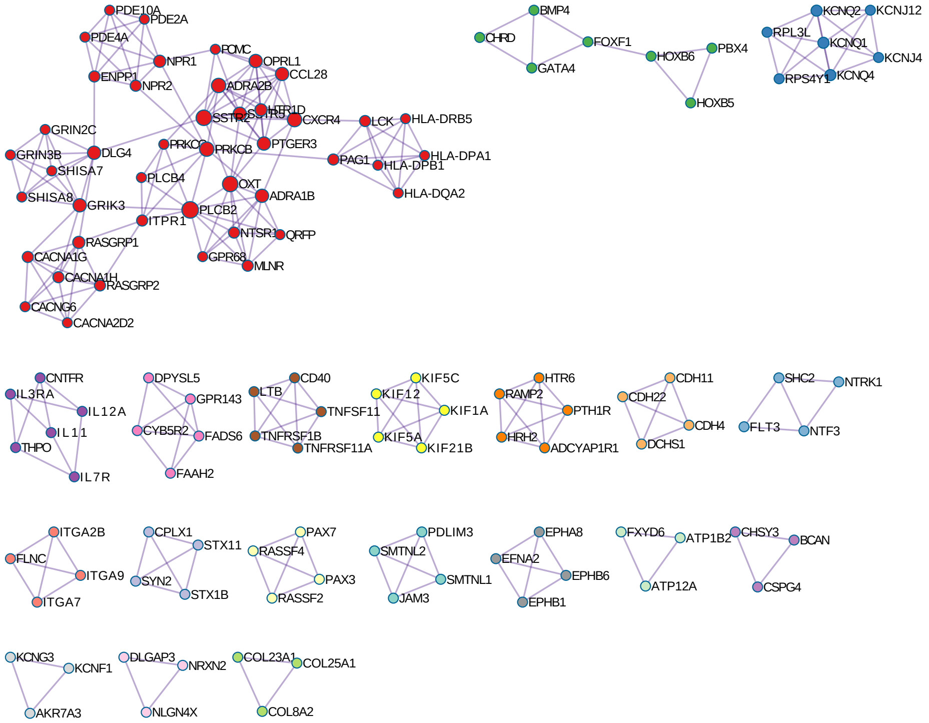

PDE10A
PDE2A
PDE4A
POMC
OPRL1
NPR1
CCL28
ENPP1
NPR2
ADRA2B
HTR1D
SSTR2
CXCR4
LCK
HLA-DRB5
GRIN2C
PRKCG
PRKCB
PTGER3
GRIN3B
DLG4
PAG1
HLA-DPA1
SHISA7
PLCB4
HLA-DPB1
OXT
SHISA8
GRIK3
ADRA1B
HLA-DQA2
PLCB2
ITPR1
NTSR1
QRFP
RASGRP1
CACNA1G
GPR68
MLNR
CACNA1H
RASGRP2
CACNG6
CACNA2D2
BMP4
CHRD
FOXF1
GATA4
HOXB6
PBX4
HOXB5
KCNQ2
KCNJ12
RPL3L
KCNQ1
KCNJ4
RPS4Y1
KCNQ4
CNTFR
IL3RA
IL12A
IL11
THPO
IL7R
DPYSL5
GPR143
CYB5R2
FADS6
FAAH2
CD40
LTB
TNFSF11
TNFRSF1B
TNFRSF11A
KIF5C
KIF12
KIF1A
KIF5A
KIF21B
HTR6
RAMP2
PTH1R
HRH2
ADCYAP1R1
CDH11
CDH22
CDH4
DCHS1
SHC2
NTRK1
FLT3
NTF3
ITGA2B
FLNC
ITGA9
ITGA7
CPLX1
STX11
SYN2
STX1B
PAX7
RASSF4
PAX3
RASSF2
PDLIM3
SMTNL2
SMTNL1
JAM3
EPHA8
EFNA2
EPHB6
EPHB1
FXYD6
ATP1B2
ATP12A
CHSY3
BCAN
CSPG4
KCNG3
KCNF1
AKR7A3
DLGAP3
NRXN2
NLGN4X
COL23A1
COL25A1
COL8A2

MCODE1
MCODE2
MCODE3
MCODE4
MCODE5
MCODE6
MCODE7
MCODE8
MCODE9
MCODE10
MCODE11
MCODE12
MCODE13
MCODE14
MCODE15
MCODE16
MCODE17
MCODE18
MCODE19
MCODE20

created by
http://metascape.org
